# NetworkViewer: visualizing biochemical reaction networks with embedded rendering of molecular interaction rules

**DOI:** 10.1186/1752-0509-8-70

**Published:** 2014-06-16

**Authors:** Hsueh-Chien Cheng, Bastian R Angermann, Fengkai Zhang, Martin Meier-Schellersheim

**Affiliations:** 1 Laboratory of Systems Biology, National Institute of Allergy and Infectious Diseases, National Institutes of Health, Building 4, 4 Memorial Drive, 20892 Bethesda, USA; 2 Department of Computer Science, University of Maryland, A.V. Williams Building, University of Maryland, 20742 College Park, USA

**Keywords:** Visualization, User interface, Protein reaction networks, Cellular signaling, Rule-based modeling

## Abstract

**Background:**

Network representations of cell-biological signaling processes frequently contain large numbers of interacting molecular and multi-molecular components that can exist in, and switch between, multiple biochemical and/or structural states. In addition, the interaction categories (associations, dissociations and transformations) in such networks cannot satisfactorily be mapped onto simple arrows connecting pairs of components since their specifications involve information such as reaction rates and conditions with regard to the states of the interacting components. This leads to the challenge of having to reconcile competing objectives: providing a high-level overview without omitting relevant information, and showing interaction specifics while not overwhelming users with too much detail displayed simultaneously. This problem is typically addressed by splitting the information required to understand a reaction network model into several categories that are rendered separately through combinations of visualizations and/or textual and tabular elements, requiring modelers to consult several sources to obtain comprehensive insights into the underlying assumptions of the model.

**Results:**

We report the development of an application, the *Simmune NetworkViewer*, that visualizes biochemical reaction networks using iconographic representations of protein interactions and the conditions under which the interactions take place using the same symbols that were used to specify the underlying model with the *Simmune Modeler*. This approach not only provides a coherent model representation but, moreover, following the principle of “overview first, zoom and filter, then details-on-demand,” can generate an overview visualization of the global network and, upon user request, presents more detailed views of local sub-networks and the underlying reaction rules for selected interactions. This visual integration of information would be difficult to achieve with static network representations or approaches that use scripted model specifications without offering simple but detailed symbolic representations of molecular interactions, their conditions and consequences in terms of biochemical modifications.

**Conclusions:**

The Simmune NetworkViewer provides concise, yet comprehensive visualizations of reaction networks created in the Simmune framework. In the near future, by adopting the upcoming SBML standard for encoding multi-component, multi-state molecular complexes and their interactions as input, the NetworkViewer will, moreover, be able to offer such visualization for any rule-based model that can be exported to that standard.

## Background

In the field of cell biology, computer simulations of intra- and inter-cellular signaling processes permit researchers to explore the validity of hypotheses about the mechanisms that cells use to process stimuli they receive from their environments. Such stimuli can, for example, consist of the binding of a hormone to cellular surface receptors and may initiate a series of reactions inside the cell, eventually leading to a change of cell state, directed movement, its proliferation or death.

Software tools such as Cytoscape [1], Osprey [2] and VisANT [3] are widely used to analyze genetic networks and pathways, providing a variety of filtering methods and visualizations. Typically, the networks being analyzed with these tools consist of relatively simple nodes (e.g. genes) that are connected by lines if they represent entities that, in some way, show correlated behavior.

Other methods have been developed specifically for visualizing cell biological protein reaction networks where the nodes frequently have some additional inner structure and the links between them indicate biochemical processes, for instance when representing multi-molecular complexes and their reactions. The Systems Biology Graphical Notation (SBGN) [4] project, for example, provides a well documented standard for visualizing biological processes, including protein interactions. It offers three different views visualizing aspects such as the flow of information (activity flow), entity relationship diagrams and can provide diagrams giving information about the sequence of biochemical modifications components in the network undergo.

Molecular Interaction Maps (MIMs) [5,6] aim at combining as much information as possible in a single diagram. However, a comprehensive visualization of all reactions, involved binding sites and molecular states permitting those reactions and how they are modified in the course of the reactions is possible only for rather small networks. The reason is that users have to simultaneously trace multiple lines to infer reaction requirements which can render the process of parsing complex interaction diagrams cumbersome.

Much of the complexity of reaction networks arises from the fact that molecules and pairwise molecular interactions frequently participate as elements in several multi-molecular complexes. Reducing model definitions back to this fundamental level, rule-based modeling approaches offer concise ways to specify molecular interactions, their conditions and consequences [7-9] and several iconographic representations of such rules have been suggested [10-12].

Using the rule-based BioNetGen language (BNGL) [7], the visualization tool RuleBender [13] addresses the conflict between readability and completeness by linking a contact map depicting possible interactions between molecular binding sites with BNGL code elements of the full rule set from which the contact map is derived. This represents a significant step forward but comes at the cost that the visualization itself contains only part of the information. Interactions and states have to be selected to access additional information via the textual mode of BNGL. Users are thus required to learn the model description language, which may impede communication between modeling experts and experimental biologists not familiar with BNGL. Another rule-based approach, Extended Contact Maps [14], provides more detailed information but also follows the strategy of omitting reaction aspects in order to increase readability. The additional information that is necessary to understand a particular reaction can be retrieved from accompanying textual explanations of the labels in the map.

The rxncon software [15] takes a modular approach to visualizing reaction networks at various levels of complexity by separating *elemental reactions* - that take molecular complexes as input and modify them through reactions - from *contingencies* that specify under which conditions these reactions may occur. Based on combining the information in these two categories in various ways, the rxncon software can generate several different pathway visualizations, including SBGN based graphs, with varying degree of completeness with regard to rendering the assumptions of the underlying models. This modular approach results in highly efficient visualizations of various aspects of interaction networks but to fully access the conditions for and the consequences of reactions, one has to consult *reaction graphs* or *reaction lists* together with *contingency lists*.

The approaches discussed so far have in common that their network visualizations either become very complicated as models grow or (for the rule-oriented approaches) that they separate the display of molecular reactions from the information regarding the conditions under which those reactions occur.

Here, we report the development of the *Simmune NetworkViewer* that takes advantage of the visual language of the *Simmune Modeler* to integrate these two aspects into a single display. Both, the Modeler and NetworkViewer, are part of the *Simmune* package [8,16,17], a framework of computer programs that allows researchers to build, simulate and analyze quantitative models of cellular signaling processes. The Modeler permits defining iconographic representation of molecular complexes, their interactions and the initial and resulting states of the interacting molecules. Based on these inputs, Simmune automatically builds complete network representations and permits users to perform simulated experiments of cellular systems containing these networks. In contrast to other approaches, the *native* representation of the reaction rules here is thus a visual, symbolic one. This allows the NetworkViewer to provide a highly efficient method for rendering protein reaction networks, addressing the preeminent challenge for network visualization, namely combining high-level overviews with details provided on-demand.

The NetworkViewer first creates a general overview showing all user-defined molecular complexes and the features determining their possible states (for example the potential to carry phosphorylations or to assume specific conformations). These complex *prototypes*, or, in the language of Simmune, *complex species*, that do not carry any particular states are linked by the biochemical network resulting from the structural interaction possibilities among their molecular binding sites. Within this display, reactions can be selected, prompting the NetworkViewer to visually indicate the particular states the participating complexes are in when the reactions occur and what their resulting states are, thus providing complete specifications of reaction rules embedded into network maps. Additional functionality permits searching for reaction rules that meet user-defined criteria, such as belonging to a certain reaction category or including certain types of reacting species. Importantly, the search results are presented as an overlay on top of the global network view, thereby presenting the reactions within their biochemical contexts. Since model specification in Simmune is based on iconographic symbols the software is rather easily accessible to non-theorists. Until now, there had been, however, no tool for the visualization of the resulting networks taking full advantage of Simmune’s approach for visually encoded molecular interactions.

## Implementation

### Simmune framework

The Simmune framework consists of a set of modeling tools including an application for specifying molecular properties and interactions (the Simmune Modeler), a cell morphology design application, and a simulator. Using the graphical interface of the Simmune Modeler [12], researchers define molecules, their components (sub-domains) and binding sites as well as interactions between such binding sites and how they depend on the states of the interacting molecules. In the following we briefly introduce the visual language and the terminologies in the Simmune Modeler using a simple ligand-receptor reaction as an example. In this example, a receptor is embedded into the cytoplasmic membrane and consists of an extracellular and an intracellular domain. When the extracellular domain binds to its ligand, the intracellular domain switches its state from inactive to active, allowing it to interact with other molecules inside of the cell, thereby initiating an intracellular signaling process.

**Figure 1:**
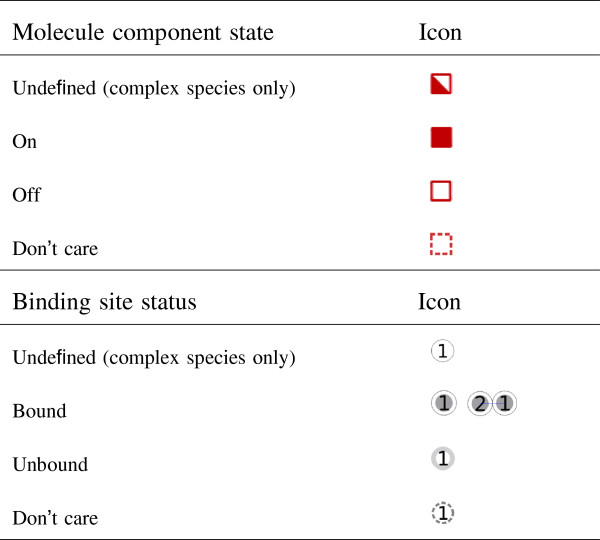
**List of icons.** The icons used for different molecule component states and binding site statuses.

In the absence of a globally accepted standard for visual representation of molecules, their components and binding sites as well as multi-molecular complexes, the Simmune Modeler allows users to create such representations using simple ellipsoidal shapes. To represent different molecule component states and binding site statuses Simmune uses the icons listed in Figure 1. Molecule components can be assigned several squares representing state variables that can be “on”, “off” or “don’t care” and may represent, for example, phosphorylations or conformational states, depending on the nature of the molecules and their interactions. Circles represent binding sites (with site indexes displayed inside the circles) and blue lines connect pairs of bound sites (filled circles). Future releases of the Simmune Modeler and the NetworkViewer will permit using icon libraries based on existing visualization approaches such as the one used in the STKE database of signaling pathways (http://stke.sciencemag.org/cm/), or the SBGN style [18].

#### Complex species and complexes

A *complex species* comprises a specific set of structurally identical *complexes* that are constructed with the same set of molecules and binding site interactions. However, the complexes within a species can differ with regard to the states of their components. Thus, whereas a complex species can be viewed as a prototype describing a particular set of complexes that are structurally identical, a complex is an “instance” of the complex species it belongs to. This hierarchy of providing structural and state-specific information about molecular complexes is fundamentally important for the ability of the Simmune NetworkViewer to generate concise reaction network visualizations. Note that in the rest of this text “species” is used interchangeably with “complex species”.

#### Reaction rules

Simmune builds reaction networks automatically from the specification of bi-molecular interactions that are described as reaction rules. Depending on their characteristics, reaction rules belong into one of the three categories: complex association, complex dissociation and complex transformation. Note that reaction rates are crucial to the specification but omitted here for simplicity.

**Figure 2:**
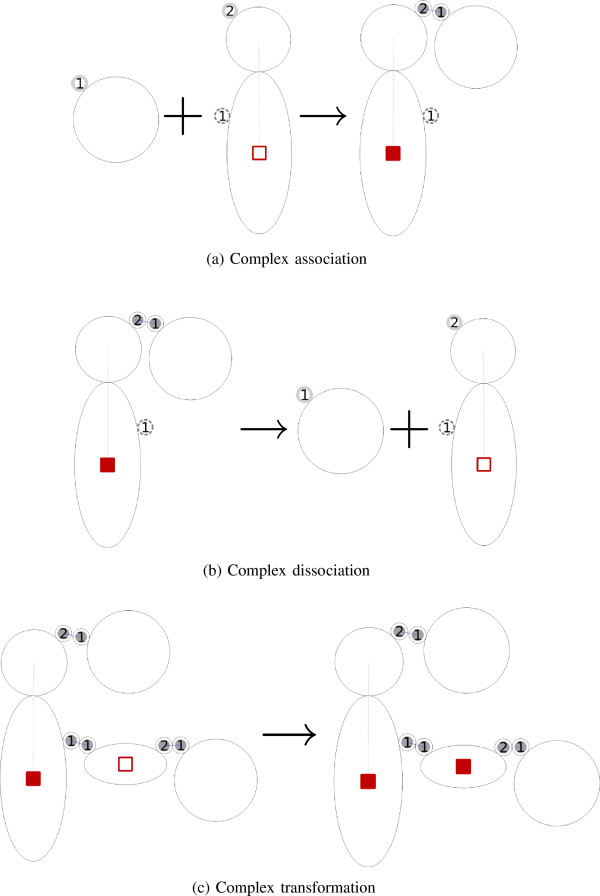
**Complex association, dissociation and transformation. (a)** A complex association where the two complexes on the left-hand side, Ligand and Rec inactive, bind and produce a Ligated Receptor complex. **(b)** A complex dissociation where Ligated Receptor on the left-hand side splits into a Ligand and a Rec inactive complex. **(c)** A complex transformation where the reacting complex LigReg_Gab_GDP transforms into the product complex LigRec_Gabg_GTP.

Exemplifying a reaction of the association type, receptor ligation is a reaction where a ligand binds a receptor, inducing a change in the receptor’s conformational and functional state. Figure 2a shows a complex association where the Ligand binds the extracellular domain of the Rec inactive complex and produces the Ligated Receptor complex. The receptor’s intracellular molecule component state changes from “off” to “on”, reflecting the change in the receptor’s state from inactive to active. Note that for the definition of reactions we refer to “complexes” even if they consist of single molecules.

Ligand dissociation is a reaction that dissociates the ligand from the receptor by removing the bond between them. Figure 2b shows a complex dissociation where the reacting complex Ligated Receptor breaks into two product complexes, the Ligand and the Rec inactive complex, after the bond between the receptor and the ligand is dissolved. The receptor’s molecule component state changes from “on” to “off” to reflect its deactivation.

To include a molecule transformation reaction, we allow the intracellular domain of the activated (ligand-bound) receptor to interact with a G-protein and enzymatically catalyze the replacement of Guanine Diphosphate (GDP) at the G-proteins’ G *α* subunit through Guanine Triphosphate (GTP). Figure 2c shows the visual representation of this complex transformation mediated by the receptor that changes the G *α* state from GDP to GTP. This is reflected by the switch of the “GTP” state (i.e. represented by the square in the horizontal ellipse depicting G *α*) from “off” to “on”.

### Network graph description

The Simmune NetworkViewer generates and visualizes a network graph, which is a directed bipartite graph composed of two categories of nodes: complex species nodes and intermediate nodes, the latter representing reactions. The total number of nodes in the graph thus equals the number of complex species plus the number of reactions.

**Figure 3:**
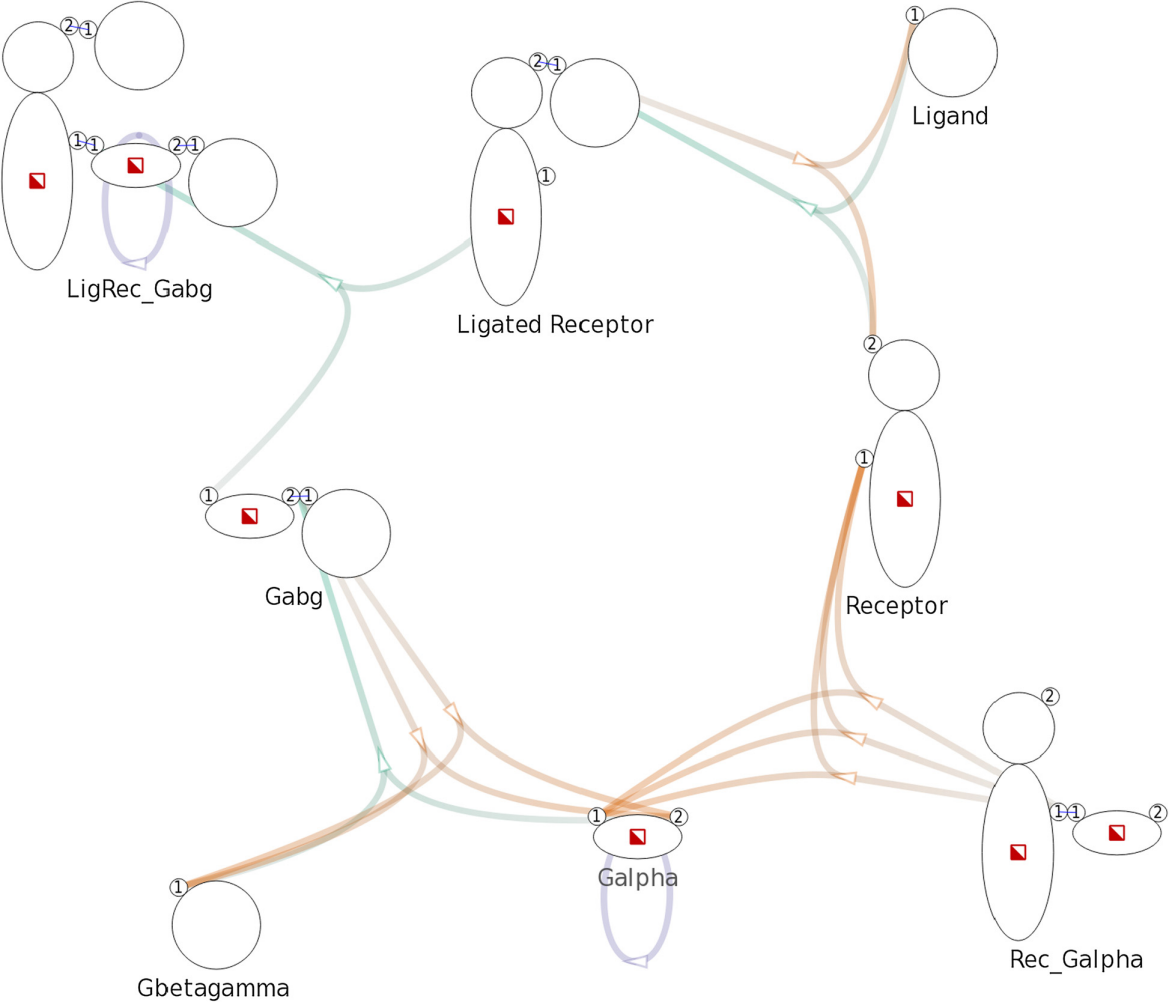
**Overview of a G-protein network.** Overview of a G-protein network with 19 nodes and 29 edges created with a model that consists of 8 complex species and 11 complex reactions. Complex species nodes are displayed with the iconographic representation used in the Simmune framework. Intermediate nodes are displayed as small arrows indicating direction of reactions.

In the network graph there exists an edge between an intermediate node and a species node if and only if the corresponding reaction involves, as reactant or product, a complex of the corresponding species. If the involved complex is a reactant (e.g. in the left-hand side of the reaction), the edge goes from the species node to the intermediate node. If that complex is a product (e.g. in the right-hand side of the reaction scheme), then the edge goes from the intermediate node to the species node.The example G-protein model encompasses eight complex species and eleven reactions, including those mentioned previously in Figure 2. The corresponding Figure 3 shows a network graph with 19 nodes and 29 edges. We will describe the layout and visual design in detail later.

To optimize the efficiency of displaying network information the viewer uses two main layout principles: 

1. Create a node for each complex species instead of each complex with specific biochemical properties.

Creating nodes for all biochemically (as opposed to structurally) distinct complexes and linking them through arrows indicating reactions would frequently generate an overwhelming number of nodes in the network graph with severely limited readability and strong node overlap. Since complexes of the same species are merely different in the molecule component states and binding site statuses we can, instead, present only the complex species within the network overview, and provide complex- and reaction-specific information upon user request.

2. Introduce intermediate nodes to represent reactions.

In principle, reactions could be indicated as edges between complex species nodes. Doing so would, however, result in confusing edges when there are multiple reaction rules between a pair of complex species. This is quite a common situation since pairs of molecular complexes may have multiple interaction possibilities that are modulated by their biochemical properties.

### The simmune NetworkViewer

In the following we describe in detail the features and design elements of the NetworkViewer.

#### Node representation

We display complex species nodes using the iconographic representation used in the Simmune modeling framework, thereby providing a concise and consistent visualization. The name of a species is shown under the corresponding species node. We use small arrows to represent intermediate nodes functioning as reaction handles. The arrows also serve as indications of the direction of reactions. See Figure 3 for an example.

#### Edge representation and layout

We use different hues to distinguish types of reactions and variation in color saturation (i.e. from less saturated to more saturated) to indicate the direction of edges. As a default, we use green for complex associations, orange for complex dissociations, and purple for complex transformations. See Figure 3 for an example. However, users can freely specify colors for different types of reactions.

A highlighted edge has greater opacity and width. The tool tip on an edge shows the reaction rate of the corresponding reaction.

Each edge is rendered as a Bézier curve. Edges that represent complex associations/dissociations have one of their endpoints pointing to the binding sites involved in related reactions. Note that complex transformations do not involve any binding sites, therefore related edges point to the center of species nodes.

For example, in Figure 3, the species node Receptor has two binding sites. Five edges, representing five reactions, connect the species node Receptor: three edges point to the first binding site and two edges point to the second.

#### Network layout

The NetworkViewer provides three network layout types: non-hierarchical layout, level-based layout and circular layout. Whereas the non-hierarchical layout provides a general overview of networks, exploiting the hierarchy in networks and reorganize network layout accordingly is useful in creating meaningful visualization. Similar to the orderly MIMs proposed in [19], we construct level-based and circular layout based on the hierarchy generated after defining a reference point in the network. Users may switch among different layouts depending on the analysis they wish to perform.

##### Non-hierarchical layout

We use the NEATO [20] layout algorithm of Graphviz [21] to generate a positional layout for the nodes in the network. After experimenting with different overlap removal techniques available in Graphviz, we choose to eliminate overlaps by incorporating overlap removal constraints into the layout algorithm. A non-hierarchical layout of the network graph created from the G-protein model is shown in Figure 3.

##### Level-based layout

**Figure 4:**
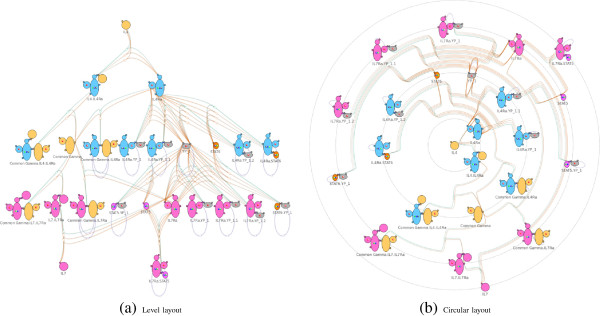
**Level-based and circular layout of a cytokine model.** After specifying the reference species IL4, the network can be reorganized using level-based and circular layout. **(a)** The corresponding reference node IL4 is placed at the top level while the other nodes are arranged with respect to their distances to the reference node. **(b)** The corresponding reference node IL4 is placed at the center while the other nodes are arranged with respect to their distances to the reference node.

In the level-based layout, nodes are arranged into levels with respect to their distances to the user-selected reference complex species node. Nodes with smaller distances (defined as the minimal number of reactions that lead from a complex to the reference node) are positioned closer to the top of the layout. The level layout is generated with the help of the DOT [22] layout algorithm of Graphviz. Figure 4a shows the level layout of a cytokine signaling model, incorporating receptors and downstream effectors for IL4 and IL7, with a reference species node IL4. The two cytokines, IL4 and IL7, and their respective receptors can be easily differentiated by color. The result of the level-based layout automatically separates the interacting species by the type of cytokines – those interacting with IL4 on the top and those interacting with IL7 at the bottom.

##### Circular layout

In the circular layout, the reference complex species node is fixed at the center and the rest of the nodes are arranged on concentric circles around this center. Similar to the criteria used in the level-based layout, nodes with smaller distances are positioned closer to the center (i.e. on concentric circle with smaller radius). We calculate the position of nodes in the circular layout with a conversion from Cartesian to polar coordinate given the result of the level-based layout. See Figure 4b for an example of the circular layout of the cytokine model with cytokine IL4 and its interacting species closer to the center, and cytokine IL7 and its interacting species on the periphery.

Passing estimates of node sizes to Graphviz allows the layout algorithms to minimize node overlap. Users can resolve residual overlap manually by adjusting the positions of nodes. In the models we tested, we found that users can resolve overlap in a short time.

The NetworkViewer saves the manually-adjusted layout as well as other visual attributes such as edge width in an auxiliary file, which, when provided along with the model file, guides the NetworkViewer to generate identical visualization using the stored configuration. We note that the ability of saving the changes to the automatically generated visualization may also help to convey information (e.g. for emphasizing certain network sections) as part of remote collaborations.

#### Tree view and reaction list

In addition to the aforementioned graphical network display, we show the species-complex hierarchy in a tree view. In another panel, we list all reactions grouped into the three reaction types (associations, dissociations, transformations). Selections performed in the tree view and reaction list are carried over into the graphical network display.

#### Filtering

The NetworkViewer facilitates locating relevant complexes and/or complex species in the tree view by allowing users to filter by either (1.) complex name or (2.) component molecules. 

**Figure 5:**
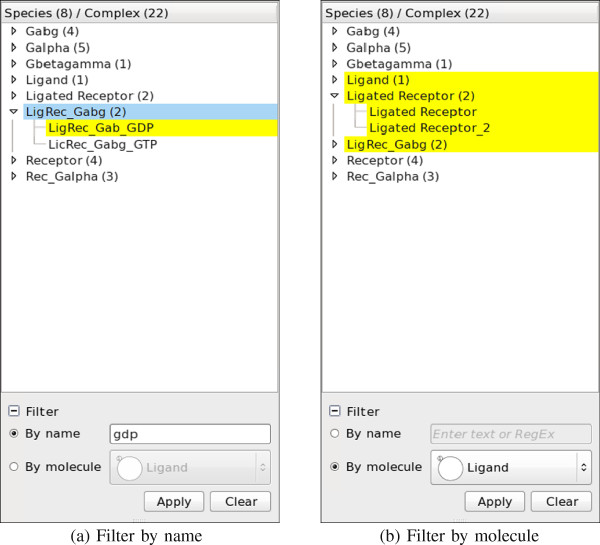
**Filtering by name and molecule. (a)** With the search term “gdp”, the matching complex LigRec_Gab_GDP is highlighted in yellow. The non-matching complex species LigRec_Gabg has a matching complex and is therefore colored in light blue. **(b)** The complex species and complexes that contain the molecule Ligand are highlighted in yellow.

1. The NetworkViewer highlights complexes and complex species whose names contain the specified term in yellow. When a complex species does not contain the term in its name but one of its child complexes does, the complex species is shown in light blue to indicate that it has at least one matching child complex that might be hidden in the collapsed list. See Figure 5a for an example of filtering by the term “gdp”.

2. The NetworkViewer highlights complexes and complex species that contain the specified molecule in yellow. For example, Figure 5b shows that only three complex species: Ligand, Ligated Receptor and LigRec_Gabg contain the molecule Ligand. Note that a complex contains a molecule if and only if its species contains that molecule too.

#### User interaction

After the initial automated layout process, the network graph (see the example shown in Figure 3 and Figure 4) provides an overview of the network model that offers an accessible abstraction at species level. Different types of specific information are presented upon user request.

Within the layout, a complex species usually interacts only with complex species nearby. Users can zoom in and move to specific regions of interest. To focus on a complex species it can be selected by either clicking the complex species node in the network display or the corresponding item in the tree view. The selected complex species and the reactions in which it is involved are highlighted.

**Figure 6:**
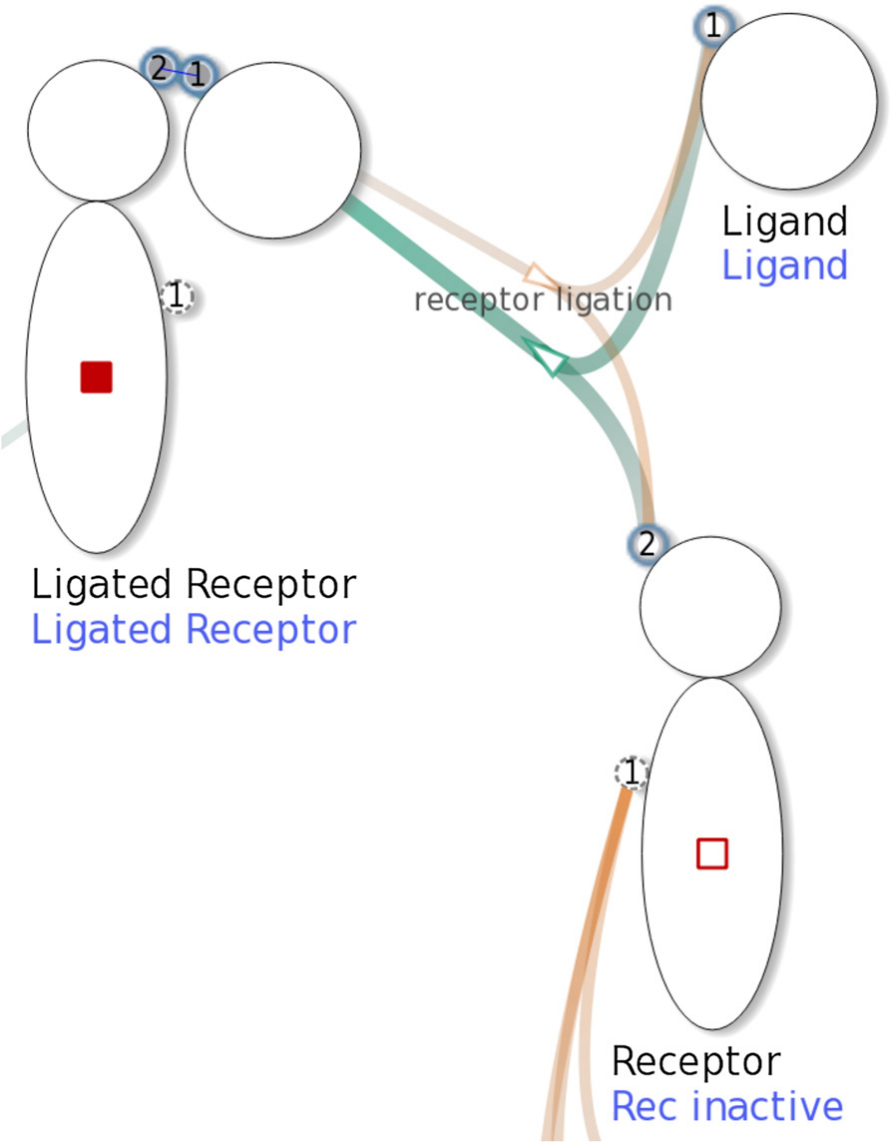
**Selecting the complex association **receptor ligation**.** The three complex species nodes show the three involved complexes, Ligand, Rec inactive and Ligated Receptor, after the complex association receptor ligation (shown in Figure 2a) is selected.

**Figure 7:**
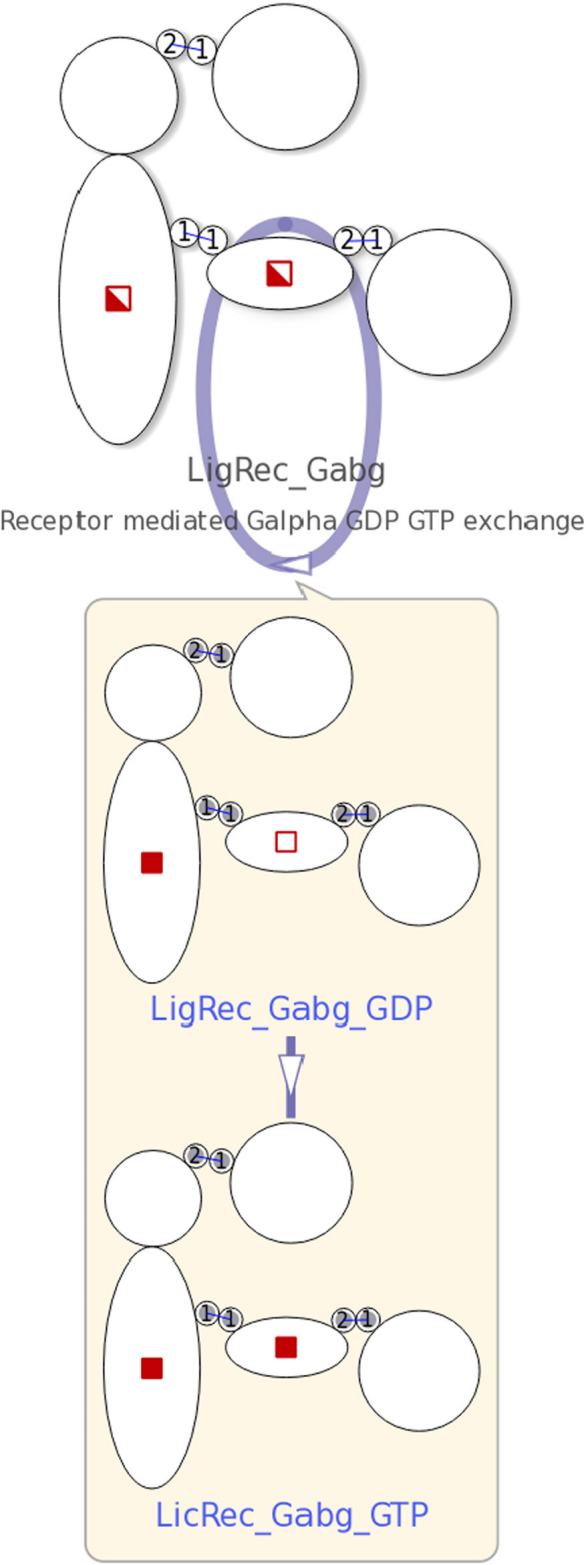
**Rendering of user-requested details about the complex transformation **Receptor mediated Galpha GDP GTP exchange**.** A hovering frame shows the two involved complexes, LigRec_Gab_GDP and LicRec_Gabg_GTP, after the complex transformation Receptor mediated Galpha GDP GTP exchange (shown in Figure 2c) is selected.

Reactions can be selected by clicking intermediate nodes (representing the reactions) in the network, or items in the reaction list. The NetworkViewer indicates selected reactions by highlighting all the related edges.When the selected reaction is a complex association or a complex dissociation, the involved complex species nodes are depicted with their molecular states and binding site statuses according to the specified reaction rule. For example, the binding sites that prior to a selected association were unbound are now linked through bonds. The names of the complexes are added to the labels in blue beneath the name of the species. See Figure 6 for an example. If the selected reaction is a complex transformation, a hovering frame, as shown in Figure 7, shows the initial and product complex.

A typical user query consists of identifying which reactions a particular complex is involved in. After the complex has been selected in the tree view it is highlighted in network visualization along with its reactions.

**Figure 8:**
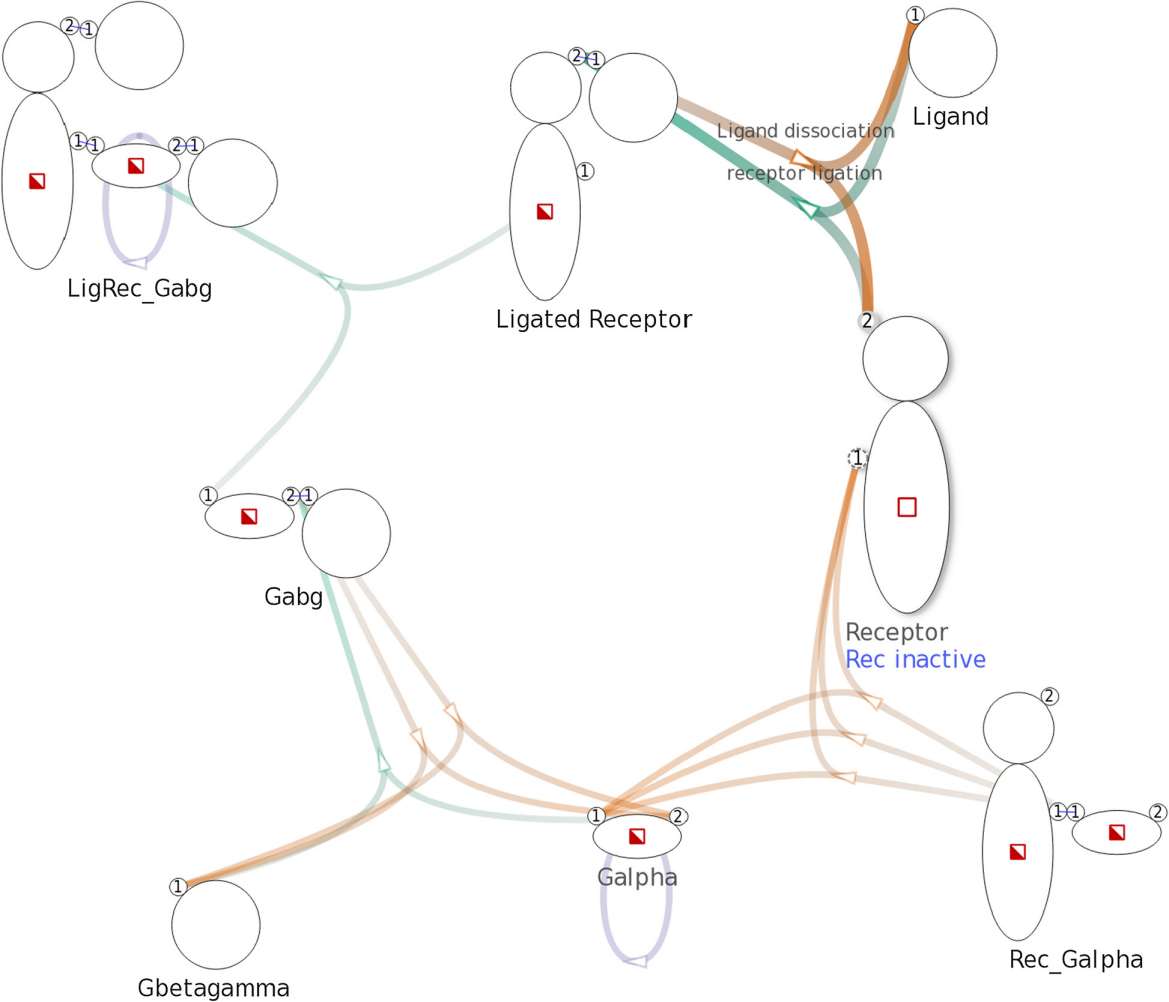
**Selecting the complex **Rec inactive**.** After selecting the complex Rec inactive, the related edges generated by the two reaction rules in Figure 2a and Figure 2b are highlighted. The visualization shows that Rec inactive participates in the two aforementioned reactions only, since other edges in the network remain unchanged.

For example, in Figure 8, after selecting the complex Rec inactive the NetworkViewer highlights two reactions, which are shown in Figure 2a and Figure 2b, that involve Rec inactive.

Searches can also be performed for complexes of a complex species that match a particular set of states. Such set of states could, for example, be combinations of phosphorylations on molecules carrying multiple phosphorylation states. The NetworkViewer finds and shows all reactions having a reactant or product complex that matches the constraint.

**Figure 9:**
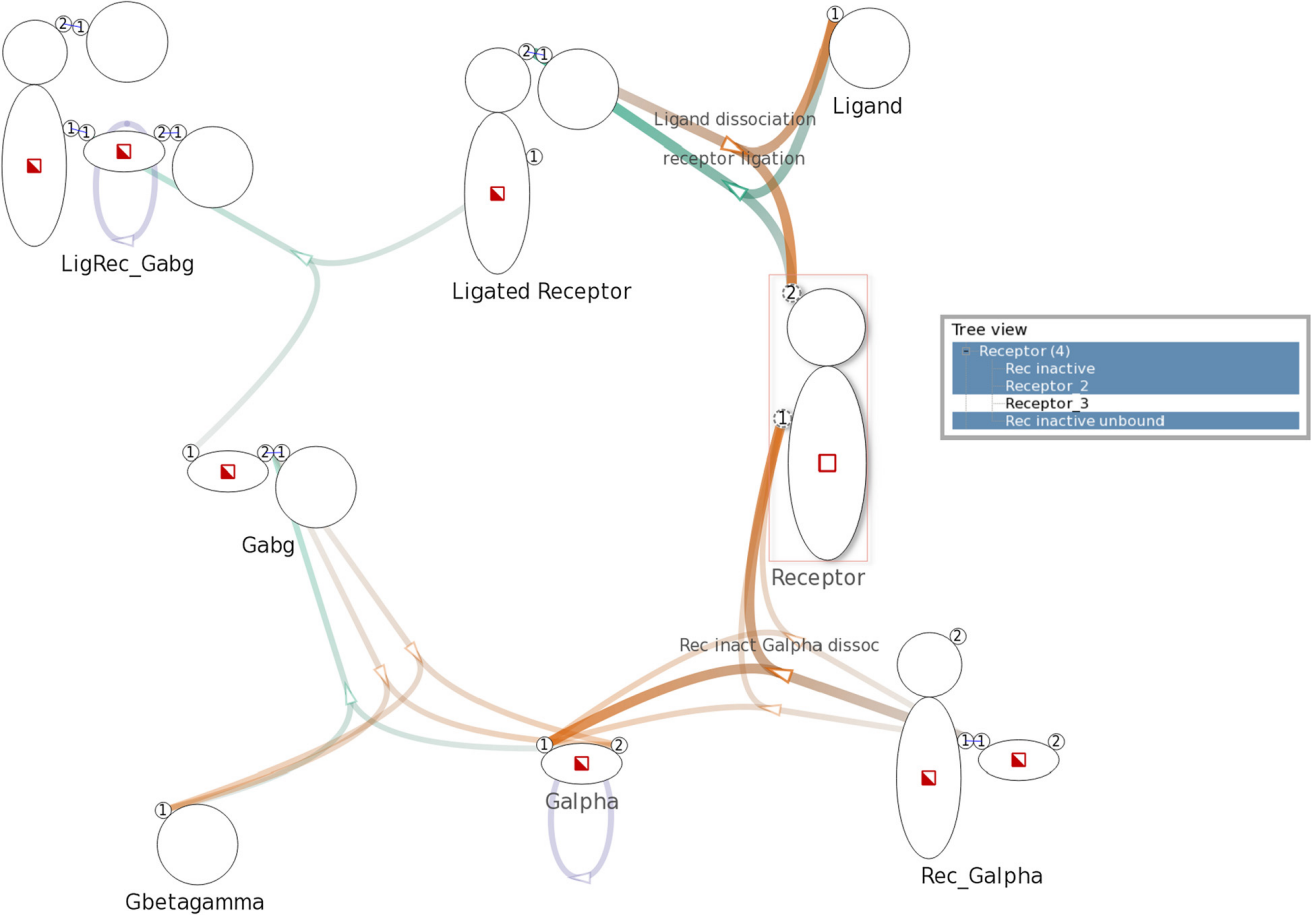
**Searching for matching complexes within complex species **Receptor**.** A search is performed on the complex species Receptor to find complexes with an “off” state in the intracellular molecule component. The edges are highlighted to show that three reactions have a reactant or product complex with an “off” state in the intracellular molecule component.

The complex species being searched is marked by a red border. Users can change the search constraint by clicking the squares that represent the states. The complexes that match the specified set of molecule component states will be selected. During the search both states “on” and “off” will match a user defined query state “don’t care”. Figure 9 shows a search on the complex species Receptor. The specified search constraint is an “off” state in the intracellular molecule component. Three complexes, Rec inactive, Receptor_2 and Rec inactive unbound, match the constraint. The matching complexes are involved in three reactions that are highlighted in the display.

### Results and discussion

To illustrate some of the capabilities of the NetworkViewer, we apply the tool to explore a model for the binding of the Epidermal Growth Factor Receptor (EGFR) to its binding partners. EGF provides proliferation, differentiation and survival signals and the membrane-bound EGF receptor is associated with several types of cancer if its expression or activation changes erroneously. The model we developed is based on the work by Hsieh et al. [23] addressing the possibility of multiple adaptors to bind to the same phosphorylated EGFR cytoplasmic (intracellular) domain simultaneously as opposed to competitively (or sequentially). Note that these constraints regarding the possible combinations of molecular interactions were obtained using coarse-grained modeling and may, thus, contain methodological artifacts. But our goal here is to illustrate the application of the NetworkViewer for visualizing networks based on interaction rules and the proposed constraints are very well suited to be implemented in a rule based model. Following [23], an EGFR cytoplasmic domain in our model has four binding sites, 992, 1068, 1148 and 1173 that, when phosphorylated at the tyrosine residues, can mediate interactions with adaptor molecules Grb2, PLC *γ*1, Stat5 and Shc. For our model, we assume that the sites are, indeed, tyrosine-phosphorylated and assign the names pY992, pY1068, pY1148 and pY1173 to the sites, where the pY stands for Tyrosine-phosphorylated. Note that a more complete model of the EGF receptor would have to take into account that the receptor undergoes ligand-induced dimerization prior to activation (phosphorylation).

**Figure 10:**
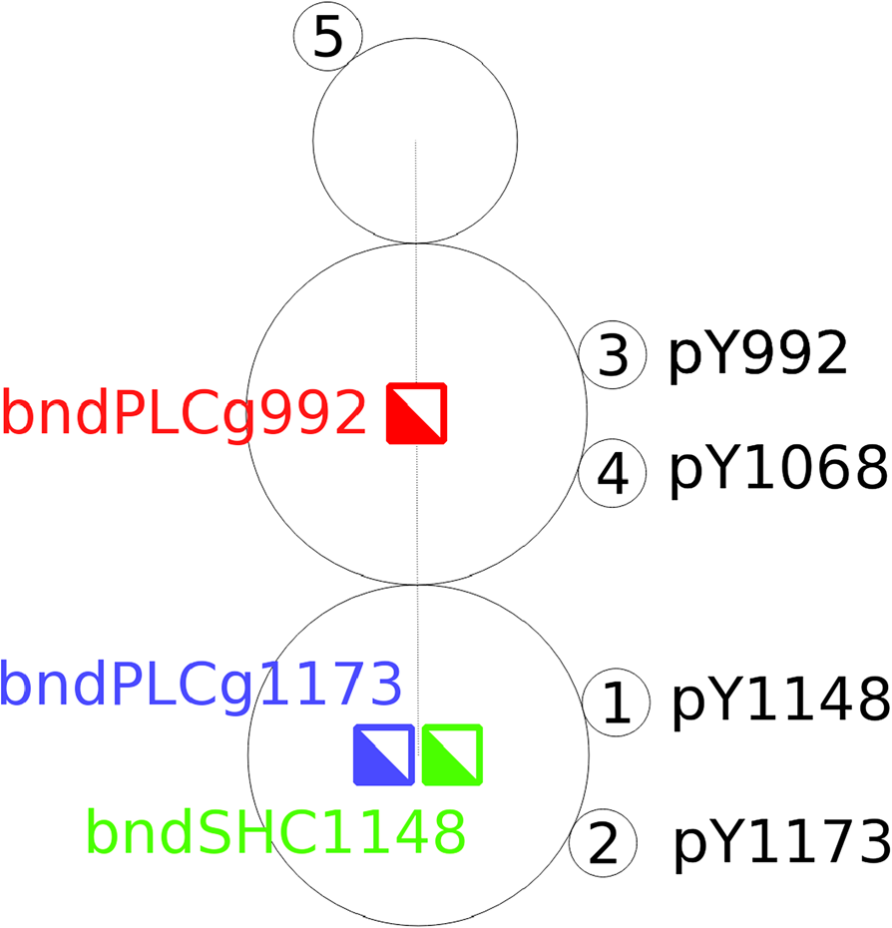
**The complex species **EGFR** and its binding sites and molecule component statuses.** The complex species EGFR has five binding sites, four of them (e.g. with indices 1 – 4) can be used to bind adaptors. Three molecule component states, “bndPLCg992”, “bndPLCg1173” and “bndSHC1148” accommodate the binding constraints reported in [23], which are described as rules defining which adaptors can bind simultaneously to the EGFR.

Stat5 and Grb2 can bind to site pY992 and pY1068, respectively. PLC *γ*1 can bind to pY992 or pY1173. Shc can bind to pY1148 or pY1173. These six interaction possibilities were translated into visually encoded reaction rules using the Simmune Modeler. In [23], the authors reported several binding constraints in this system. For example, once an adaptor PLC *γ*1 binds to pY992 or pY1173, it prevents another PLC *γ*1 from binding to the other, remaining, site. To accommodate these constraints in our model, we assigned two molecule component states “bndPLCg992” and “bndPLCg1173” to the EGFR species indicating whether a PLC *γ*1 is bound to either one of the two binding sites pY992 and pY1173, respectively. An additional state “bndSHC1148” is needed for the constraint that the binding of Shc to site pY1148 and the binding of PLC *γ*1 to site pY1173 are mutually exclusive. Figure 10 shows the representation of complex species EGFR with the aforementioned binding sites and molecule component statuses.

**Figure 11:**
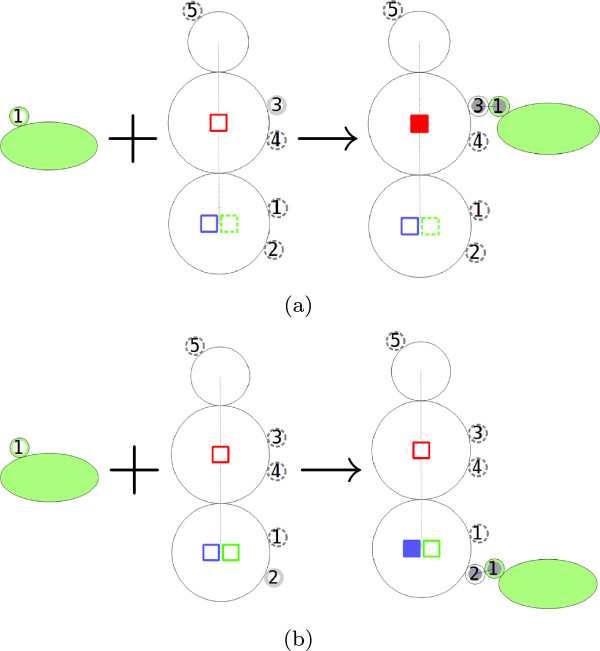
**The binding of PLC*γ*1 to EGFR. (a)** Two states “bndPLCg992” and “bndPLCg1173”, represented as red and blue squares, have to be “off” for PLC *γ*1 to be able to bind to site pY992. **(b)** All three states “bndPLCg992”, “bndPLCg1173” and “bndSHC1148”, represented as red, blue and green squares, have to be “off” for PLC *γ*1 to be able to bind to site pY1173.

The conditions for binding of PLC *γ*1 to the EGFR using the two possible sites are depicted in Figure 11. PLC *γ*1 can only bind to EGFR when both molecule component states “bndPLCg992” and “bndPLCg1173” are “off”. After ligation, the corresponding state – pY992 or pY1173, depending on which site PLC *γ*1 has bound to, switches to “on”, thereby blocking the other site for a second PLC *γ*1 molecule. As depicted in Figure 11b “bndSHC1148” must be in the “off” state to permit the binding of PLC *γ*1 to site pY1173.

**Figure 12:**
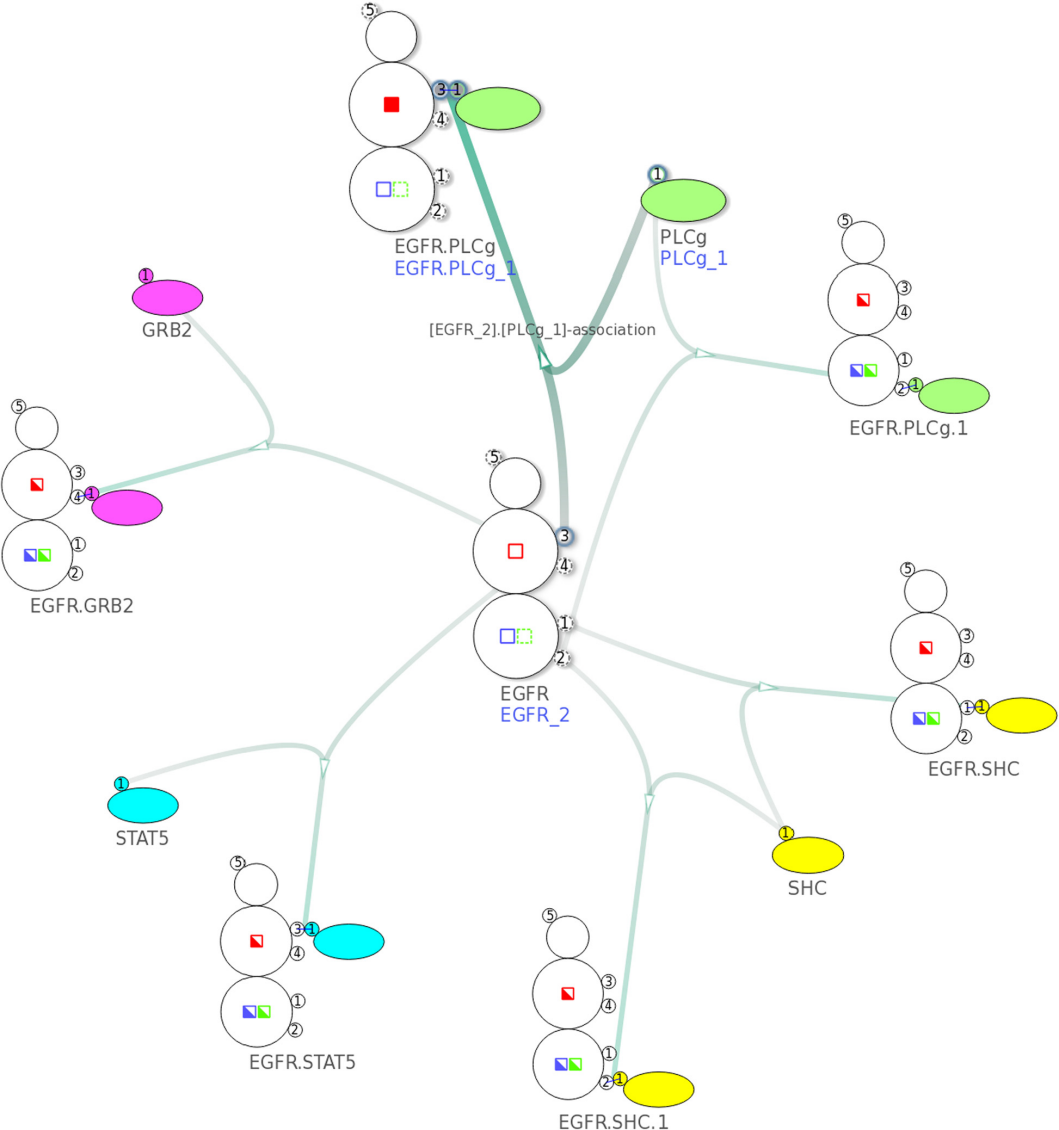
**Overview of the EGFR model **[23]**.** The network contains 17 nodes and 18 edges created from 11 complex species and 6 complex reactions. Here we select the complex association rule described in Figure 11a.

After loading the model into the NetworkViewer, the network overview in Figure 12 shows the possible reactions between the adaptors and the EGFR as well as the binding sites these reactions involve. For example, PLC *γ*1 can bind in two ways to the EGFR using two different binding sites. After selecting the corresponding intermediate node, the display shows that the binding of PLC *γ*1 to site pY992 changes the state “bndPLCg992” from “off” to “on”.

**Figure 13:**
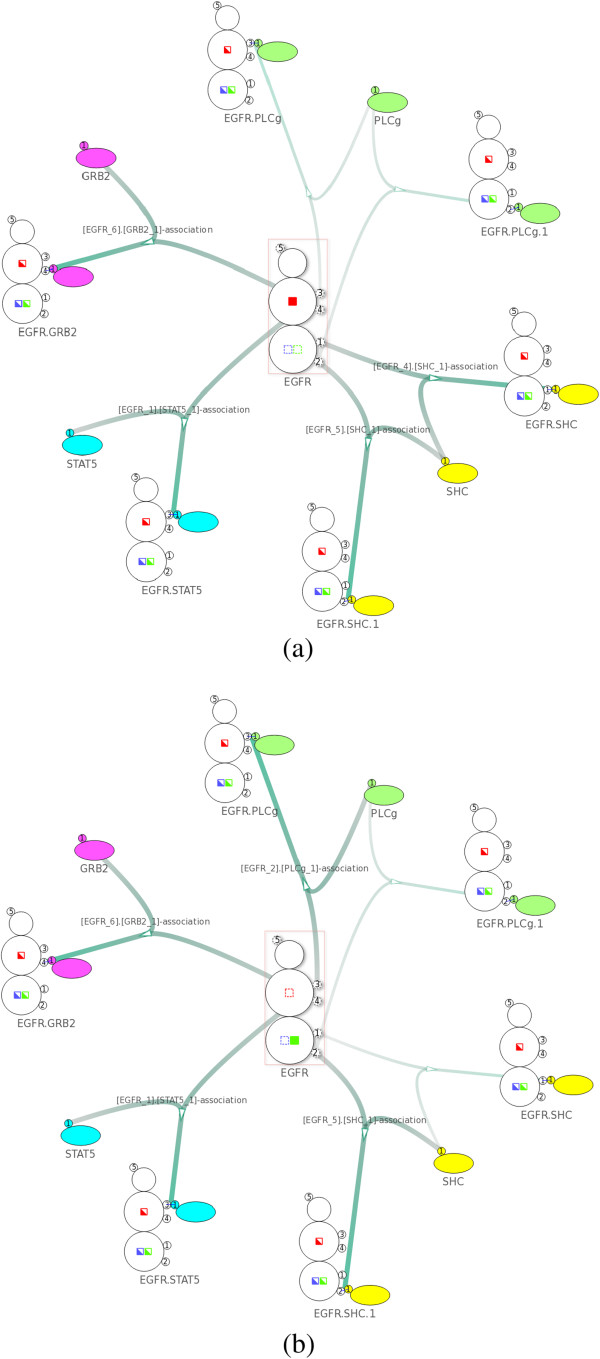
**Using the search feature to verify the binding constraints shown in Figure **11**. (a)** After specifying a search constraint where the state “bndPLCg992” is on, the visualization shows that PLC *γ*1 cannot bind to site pY992/pY1173. **(b)** After specifying a search constraint where the state “bndSHC1148” is on, the visualization shows that PLC *γ*1 cannot bind to site pY1173.

We can now use the search functionality to verify the binding constraints implemented in the EGFR model. For example, in Figure 13a, a search on the states of the EGFR shows that whenever the state “bndPLCg992” is “on”, no second PLC *γ*1 can bind to the EGFR. Similarly, in Figure 13b, whenever the state “bndSHC1148” is “on” PLC *γ*1 cannot bind to site pY1173. Moreover, Shc cannot bind to site pY1148 either when the state “bndSHC1148” is “on”. Since an “on” state of “bndSHC1148” indicates that Shc is already bound to site pY1148, it is obvious that there cannot be another Shc binding to the same site.

## Conclusions

Here, we introduced the NetworkViewer as part of the Simmune modeling framework. The NetworkViewer provides an interactive network model visualization that permits efficient exploration of models built with the Simmune Modeler using the same visual language. Exploiting the hierarchical nature of the reaction network model, the NetworkViewer creates a compact model overview, in which only the complex species and complex reactions are displayed as nodes. User interaction activates the presentation of detailed information about, for instance, the molecule component states of a complex participating in a particular reaction. The case study of a simple model of interactions among the EGFR cytoplasmic domain and its binding partners illustrates how the network overview and user interaction options of the NetworkViewer permit an efficient navigation of model components and interaction conditions, here provided as adaptor binding constraints.

Our current method for visualizing biochemical reaction networks is still incomplete in the sense that the actual rate at which a reaction is occurring not only depends on its rate constant but also on the concentrations of the reacting complexes. We will address this issue by incorporating simulation results into the network visualization. This obviously adds another level of complexity and the kind of information that will be visualized has to be selected carefully. The biologically relevant dynamical information will typically be at the level of patterns of states of molecular complexes or specific state sets and not on the structural level of complex species. Thus, displaying the complete dynamical state of a simulated model will be impractical and the viewer will have to dynamically select the most relevant aspect of information in a context dependent way.

Currently, the NetworkViewer only displays reaction networks created with the Simmune Modeler. However, Simmune will soon be able to import rule-based models encoded in the upcoming SBML3 *multi* (multi-state, multi-component) standard [24]. At that point, the NetworkViewer can be used to visualize any rule-based model generated by approaches adhering to this standard.

## Availability and requirements

**Project name**: Simmune NetworkViewer**Project home page**: http://www.niaid.nih.gov/labsandresources/labs/aboutlabs/lsb/Pages/simmuneproject.aspx**Operating system(s)**: Mac, Windows, Linux.**Programming language**: C++**License**: Downloadable from NIAID website. Download agreement.**Any restrictions to use by non-academics**: The software may not be used for commercial purposes without prior permission from the NIAID Office of Technology Development (Commercial license).

## Competing interests

The authors declare that they have no competing interests.

## Author’s contributions

H-C.C. wrote the software with the help of B.R.A. and F.Z.. M.M-S. initiated and supervised the project. H-C.C., B.R.A. and M.M-S. wrote the manuscript. All authors read and approved the final manuscript.
